# Ethnicity affects the risk factors of acute myocardial infarction and should be considered in educational programs

**DOI:** 10.3389/fcvm.2022.948028

**Published:** 2022-10-19

**Authors:** Umar Abdolah Alharbe, Hanad Hassan Alatawi, Palanisamy Amirthalingam, Sultan Mohammed Daghriri, Alanoud Abduallah Alhwiti, Tahani Saud Alenazi, Abdulelah Turki S. Al Ahmare, Sawsan A. Zaitone, Ahmed Aljabri, Ahmed Mohsen Hamdan

**Affiliations:** ^1^Department of Pharmacy, King Fahd Specialized Hospital, Tabuk, Saudi Arabia; ^2^Pharmaceutical Care Department, Almahrajan Primary Healthcare Centre, Ministry of Health, Tabuk, Saudi Arabia; ^3^Faculty of Pharmacy, University of Tabuk, Tabuk, Saudi Arabia; ^4^Department of Pharmacy Practice, Faculty of Pharmacy, King Khalid University, Abha, Saudi Arabia; ^5^Al-Dawaa Medical Services Company, Tabuk, Saudi Arabia; ^6^Department of Pharmacology and Toxicology, Faculty of Pharmacy, University of Tabuk, Tabuk, Saudi Arabia; ^7^Department of Pharmacology and Toxicology, Faculty of Pharmacy, Suez Canal University, Ismailia, Egypt; ^8^Department of Pharmacy Practice, Faculty of Pharmacy, King Abdulaziz University, Jeddah, Saudi Arabia

**Keywords:** acute myocardial infarction, cohort study, educational programs, ethnicity, risk factors

## Abstract

Acute Myocardial infarction is a non-communicable disease representing the leading cause of death in Saudi Arabia. Studying the ethnicity in its risk factors has been poorly investigated.

**Objectives:**

To compare risk factors among Saudi nationals and expatriates.

**Methods:**

A retrospective cohort study for patients admitted to the Cardiac Care Unit of one of the largest tertiary care hospitals in Tabuk diagnosed with acute myocardial infarction from September 2018 to August 2019. Risk factors were compared among groups on the basis of their ethnicity.

**Results:**

In total 18,746 patients were included. Gender and age were the predominant risk factors; Male (*p* < *0.05*) and age >50 years (*p* < *0.05*). There were significant differences between Saudis and expatriates in all measurable parameters indicating that a genetic factor contributes to the risk factors, which was proved by the significant differences between the Middle East North Africa and South Asia subgroups. Interestingly, the mean values of laboratory results were higher than Saudi populations.

**Conclusions:**

The disparity in lipid profile among the studied groups addresses the patient ethnicity should be considered during education programs for the risk factors of cardiovascular disease.

## Introduction

Saudi Arabia is classified as a high-income country with a gross income of nearly $22,000 per capita (1), and it has a very fast-growing economy. The combination of these factors causes it to suffer from the “disease of civilization” or “Lifestyle Disease.” This disease is a group of disorders that are collectively known as non-communicable disease (NCD). It includes different diseases like cancer, obesity, hypertension, acne, diabetes, etc. According to the reports from the Institute of Health Metrics and Evaluation, 16.4% of Saudi nationals are susceptible to die of an NCD and related disability-adjusted life years lost (DALYs) (2, 3). It has been reported that scarcity of physical activity and an unhealthy diet with a bad regime leads to obesity, hypertension, and diabetes (4). In spite of the national transformation program in Saudi Arabia which aims at improving the participation of the private sector, the government is still responsible for a great percentage of the healthcare expenses (5). Therefore, it is essential to concentrate on the primary and secondary prophylaxis of NCD.

Acute Myocardial infarction (AMI), a NCD, is the most common disorder of coronary heart disease (CHD) (6). Its risk factors include age, gender, obesity, blood pressure, glycaemic control, lipid profile, and smoking status (6–8). Saudi Arabia has a population of around 34 million as of 2020 (5) with 38% made up of expatriates from various countries and ethnic regions [9]. Most of the expatriates are from the Middle East North Africa (MENA) region (Egypt, Syria, Yemen, Jordan, and Sudan) and South Asia (India, Pakistan, Bangladesh, and Sri Lanka) (8, 9). Yet, disparity was noted in the risk factors of AMI among various ethnic regions. So, ethnicity should be considered in determining the risk factors for Cardiovascular Disease (CVD) (9). The Africa Middle East Cardiovascular Epidemiological study (ACE) conducted in Saudi Arabia substantiates the disparity of risk factors between Saudi populations and expatriates (10). Though some recent studies established the risk factors of AMI in the Saudi population (11–13), the disparity in risk factors for AMI between the Saudi population and expatriates is yet to be established. Therefore, the present study aims to evaluate the risk factors of AMI between the Saudi population and the expatriate population in the Tabuk region. Our research hypothesis was to pinpoint risk factors of AMI in patients of specific ethnicities.

## Methods

### Study design

A retrospective cohort study was undertaken including patients admitted with the diagnosis of AMI to the Coronary Intensive Care Unit (CICU) at King Fahd Speciality hospital in Tabuk region, Saudi Arabia during the period from September 2018 to August 2019 using a common registry program. It is a tertiary hospital and it is one of the largest hospitals in the Tabuk region, where it is the only health care system that treats STEMI and non-STIME patients in all Tabuk region. The patients were identified *via* reports from the electronic medical record systems of patients admitted to the CICU with a documented diagnosis of AMI.

#### Inclusion criteria

We selected only inpatients registered in the CICU according to the hospital policy to be referred to this unit and diagnosed with AMI from the first-day treatment in the hospital and stayed for three successive days in the hospital with daily laboratory analysis and complete demographic data and medical history.

#### Exclusion criteria

Patients transferred from other hospitals, those who did not complete 3 days of successive laboratory tests in King Fahd Speciality Hospital, or those who are of incomplete demographic data or medical history.

### Data collection

After receiving sufficient training, four investigators abstracted the data using a standardized data collection tool.

The following demographic details were collected: age, gender, nationality, Smoking status, diagnosis, past medical history, and family history. Laboratory investigations included glycaemic status (fasting blood sugar and HbA1C), lipid profile including total cholesterol (TC), triglycerides (TG), high-density lipoproteins (HDL), and Low-density lipoproteins (LDL). The ratio of TC/HDL, TG/HDL, and LDL/HDL ratio was calculated from the lipid profile. Data for Systolic and diastolic blood pressure were also obtained.

### Study outcomes

The aim of the current study is to evaluate the risk factors of AMI between the Saudi population and the expatriate population in the Tabuk region. Patients were deemed to have hypertension, diabetes, and/or dyslipidaemia according to the following:

### Hypertension

Patients with a documented history of hypertension or BP >140/90 mmHg during admission according to the European Society of Cardiology (14, 15).

### Diabetes

Patients with a documented history of diabetes or fasting blood sugar >7 mmol/L or HbA1C>7% during admission as per the American Diabetes Association (16).

### Lipid profile and ratio

Dyslipidaemia was reported if the patient had it in the past medical history or if he/She had one or more of the following abnormal lipid profiles: high total cholesterol (TC), high low-density lipoprotein (LDL), low high-density lipoprotein (HDL), or high triglyceride level (TG) according to the National Cholesterol Education Program Adult Treatment Panel III (NCEP ATP III) (16). Patients measured TC <5, TG <1.7, HDL >1.03, and >1.29 mmol/L in male and female, respectively, and LDL <2.5 mmol/L are considered normal. TC, TG, and LDL were subtracted with HDL to give TC/HDL, TG/HDL, and LDL/HDL ratios respectively. The normal values of these ratios can be seen in Table 1 (17, 18).

**Table 1:** Normal levels Cholesterol ratios (17, 18).

**Lipid ratio**	**Normal level in male**	**Normal level in female**
TC/HDL	<4.5	<4
TG/HDL	<3	<2.5
LDL/HDL	<2.5	<2

### Data analysis

Initially, the studied population was categorized as the Saudi population and expatriates. Furthermore, expatriates were again classified into South Asian and MENA region according to their geographical region. South Asia includes India, Pakistan, Bangladesh, and Sri Lanka. Meanwhile, the MENA region includes Egypt, Syria, Kuwait, Yemen, Turkey, and Sudan. Demographics and laboratory investigations were classified and analyzed as Saudi population, expatriates from South Asian, and MENA region in Table 2.

**Table 2:** Distribution of studied population according to the region/country.

**Region/country**	**Frequency *n* (%)**
Saudi Arabia	9,816 (52.36%)
South Asian countries	6,215 (33.15%)
Bangladesh	3,344 (53.80% of the South Asia Countries)
India	1,417 (22.79% of the South Asia Countries)
Pakistan	1,113 (17.90% of the South Asia Countries)
Sri Lanka	341 (5.48% of the South Asia Countries)
^*^MENA region	2,715 (14.48%)
Egypt	1,329 (48.95% of the MENA region)
Jordan	364 (13.40% of the MENA region)
Syria	201 (7.40% of the MENA region)
Yemen	565 (20.81% of the MENA region)
Sudan	256 (9.42% of the MENA region)

* MENA, Middle East North Africa.

### Statistical analysis

SPSS version 25 (SPSS, Chicago, Illinois) statistical software package was used for all the statistical analyses. The chi-square test was used to analyze the demographic variables between the groups. To compare the laboratory parameters between the study groups, Student's *t*-test was used. Two-tailed *p*-value <0.05 was considered significant.

## Results

### Distribution of demographic data between ethnicity

**Figure 1:**
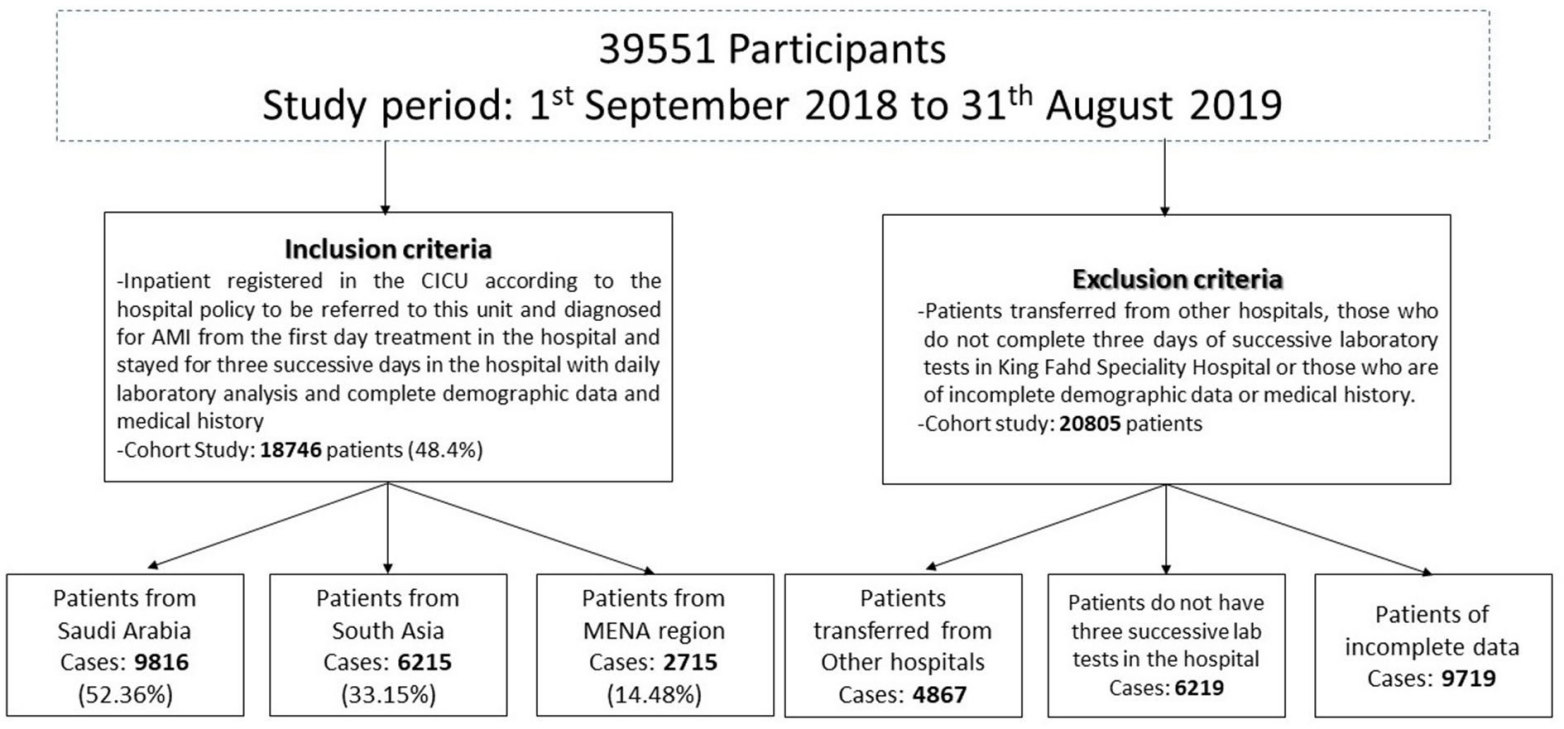
Flow chart of the cohort study.

Among 39,551 patients admitted to the Coronary Intensive Care Unit (CICU) at King Fahd Speciality hospital in the Tabuk region during the period from September 2018 to August 2019, 18,746 patients admitted with a diagnosis of acute myocardial infarction (AMI) were included in the study (about 48.4%) (Figure 1).

**Figure 2:**
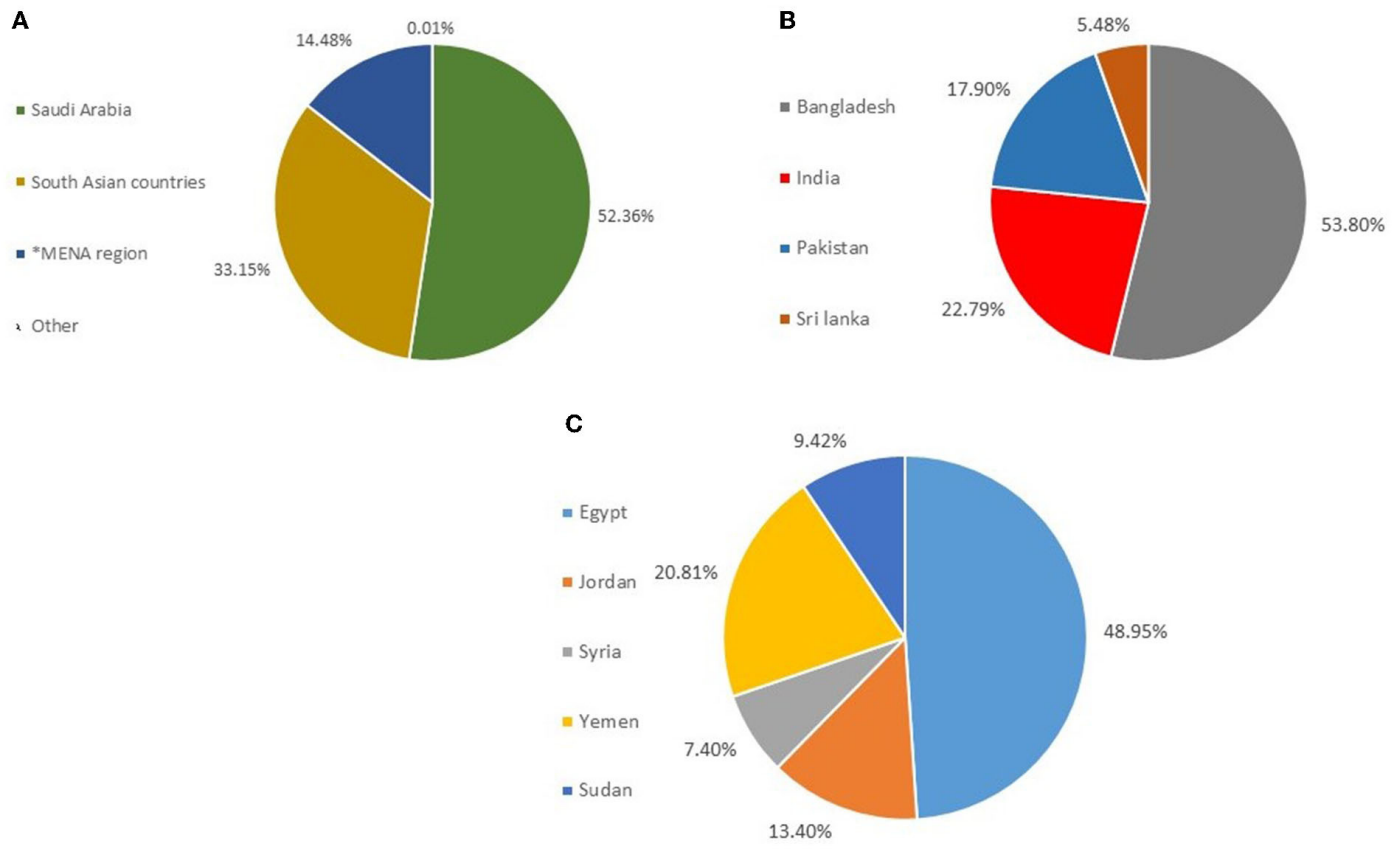
Distribution of nationalities in the studied population. **(A)** Distribution of Nationalities in Saudi Nationals, South Asian Counties, and MENA. **(B)** Distribution of Nationalities in South Asian countries **(C)** Distribution of Nationalities in MENA countries. *MENA, Middle East North Africa.

We discovered that 9,816 of the patients were from Saudi Arabia (52.36%), followed by 6,215 patients from South Asian Countries (31.15%), then 2,715 patients from the MENA region Countries (14.48%). Among the South Asian and MENA region populations, a high prevalence in the study population was noted to come from Bangladesh, 3,344 patients (53.8% of the South Asia countries) and from Egypt, 1,329 patients (48.95% of the MENA region), respectively (Table 2; Figure 2).

### The association between demographic parameters and risk factors in the studied population diagnosed with AMI

The demographics of the ethnicity and risk factor distribution in the studied population are demonstrated in Table 3. As shown in Table 3, 14,158 patients (75.52% of the total studied population in both Saudi and Non-Saudi populations) were males. It revealed that gender is a risk factor and males were more susceptible for AMI (*p*<*0.05*).

**Table 3:** The association between demographic parameters and risk factors in the studied population diagnosed with AMI.

**Characteristics**	**Study population (*n* = 18,746)**
Male, *n* (%)	14,158 (75.52%)
Age, years, mean (SD)	53.7 (8.10)
Smokers, *n* (%)	8,998 (47.99%)
**Acute myocardial infarction**
STEMI, *n* (%)	9,219 (49.17%)
NSTEMI, *n* (%)	9,527 (50.82%)
**Past medical history** ***n*** **(%)**
Diabetes	7,587 (40.47%)
Hypertension	7,546 (40.25%)
Dyslipidaemia	4,565 (24.35%)
Previous CHD	3,112 (16.8)
Family history of CHD	3,362 (17.93%)

Table 4 shows that non-Saudi males were more susceptible to AMI than Saudi malesmen. Therefore, there is an effect from ethnicity on this risk factor (*p*<*0.05*). This indicates that gender is a risk factor alongside an effect from ethnicity. A significant association was observed among the population aged >50 years with the prevalence of AMI (*p*<*0.05*) (Table 3); however, there is a significant difference between populations with age above 50 years and below 50 years of non-Saudi and Saudi populations (Table 4).

**Table 4:** The association between demographic parameters of ethnicity and risk factors in the studied population diagnosed with AMI.

**Characteristics**	**Saudi nationals Vs. expatriates** **(*****n*** = **18,746)**			**Expatriates of South Asians Vs. MENA region (*****n*** = **8,930)**		
	**Saudi nationals (*n* = 9,816) frequency (%)**	**Expatriates (*n* = 8,930) frequency (%)**	***P*-value**	**South Asians (*n* = 6,215) frequency (%)**	**Other middle east population (*n* = 2,715) frequency (%)**	***P-*value**
**Gender**
Male	8,112 (82.64)	6,046 (67.70)	**<0.05**	3,755 (60.41)	2,291 (84.38)	**<0.05**
Female	1,704 (17.35)	2,884 (32.29)		2,460 (39.58)	424 (15.61)	
**Age**
≤ 50 years	3,811 (38.82)	3,704 (41.47)	**<0.05**	2,300 (37)	1,404 (51.71)	**<0.05**
>50 years	6,005 (61.17)	5,226 (58.52)		3,915 (62.99)	1,311 (48.28)	
**Social history**
Smokers	8,998 (91.66)	4,107 (45.99)	**<0.05**	2,422 (38.97)	1,685 (62.06)	**<0.05**
Non-smokers	818 (8.33)	4,823 (54)		3,793 (61.02)	1,030 (37.93)	
**Acute myocardial infarction**
STEMI	4,668 (47.55)	4,551 (50.96)	**<0.05**	3,054 (49.13)	1,497 (55.13)	**<0.05**
NSTEMI	5,148 (52.44)	4,379 (49.03)		3,161 (50.86)	1,218 (44.86)	
**Past medical history**
Diabetes	4,534 (46.78)	3,053 (34.18)	**<0.05**	2,211 (35.57)	842 (31.01)	**<0.05**
Hypertension	4,387 (44.69)	3,159 (35.37)		2,316 (37.26)	843 (31.04)	
Dyslipidemia	2,226 (22.67)	2,339 (26.19)		1,684 (27.09)	655 (24.12)	
Previous CHD	1,177 (11.99)	1,935 (21.66)		1,052 (16.92)	883 (32.52)	
Family history of CHD	1,315 (13.40)	2,047 (22.92)	–	1,486 (23.90)	561 (20.68)	–

Chi-square test; two-tailed *p*-value < 0.05 considered as significant; MENA, Middle East North Africa. Bold values indicate the significance.

This indicates that age is a risk factor with an effect alongside ethnicity. Interestingly, both smoking and type of AMI were risk factors, and also they had significant associations with ethnicity among the studied population (*p*<*0.05*). Diabetes and hypertension were the major risk factor for AMI (40.47 and 40.25%, respectively) with a significant association with ethnicity (*p*<*0.05*). These two risk factors were followed by coronary heart disease (CHD) and dyslipidemia. Also, more than 17.9% of the studied population had a family history of CHD.

### Assessment of the measurable parameters between the saudi population and expatriates in the studied population diagnosed with AMI

#### Blood pressure

A significant difference (higher than 135) was observed between the Saudi populations and expatriates for the factor of high systolic blood pressure and normal diastolic blood pressure among the studied population, with a significant difference in the ethnic regions as well (Table 5). Saudi population showed lesser systolic blood pressure than expatriates.

**Table 5:** Assessment of measurable parameters between Saudi Nationals and Expatriates.

**Parameters**	**Saudi nationals (*n* = 9,816) mean (SD)**	**Expatriates (*n* = 8,930) mean (SD)**	***P*-value**	**Expatriates from South Asia (*n* = 6,215)**	**Expatriates from MENA region (*n* = 2,715)**	***P*-value**
Systolic blood pressure (mmHg)	133.94 (2.04)	135.93 (1.83)	**<0.05**	135.71 (1.79)	134.12 (1.36)	**<0.05**
Diastolic blood pressure (mmHg)	76.80 (0.979)	80.5 (1.48)	**<0.05**	80.08 (1.47)	81.73 (0.25)	**<0.05**
Fasting blood sugar (mmol/L)	13.10 (2.04)	11 (0.86)	**<0.05**	10.77 (0.85)	11.70 (0.24)	**<0.05**
HbA1C (%)	9.63 (1.19)	8.13 (1.06)	**<0.05**	7.88 (1.06)	9 (0.02)	**<0.05**
Total cholesterol (mmol/L)	4.10 (0.29)	4.38 (0.40)	**<0.05**	4.25 (0.37)	4.78 (0.05)	**<0.05**
Triglycerides (mmol/L)	1.46 (0.02)	2.65 (0.22)	**<0.05**	2.07 (0.20)	2.39 (0.07)	**<0.05**
LDL (mmol/L)	3.04 (0.19)	2.84 (0.13)	**<0.05**	2.79 (0.12)	2.97 (0.04)	**<0.05**
HDL (mmol/L)	1.11 (0.25)	0.66 (0.15)	**<0.05**	0.67 (0.17)	0.67 (0.12)	0.819
TC/HDL ratio	4.16 (0.20)	5.39 (0.25)	**<0.05**	5.32 (0.24)	5.64 (0.05)	**<0.05**
LDL/HDL ratio	2.94 (0.14)	3.48 (0.20)	**<0.05**	3.43 (1.19)	3.67 (0.09)	**<0.05**
TG/HDL ratio	2.72 (0.31)	3.22 (0.17)	**<0.05**	3.16 (0.16)	3.40 (0.01)	**<0.05**

Students *t*-test; two-tailed *p*-value < 0.05 considered as significant; MENA, Middle East North Africa. Bold values indicate the significance.

#### Glycaemic control

Significant difference between Saudi populations and expatriates for the factor of High fasting blood sugar (higher in Saudi population; 13.1) and HbA1C (higher in Saudi population; 9.63) were observed among the study population with a significant difference in the ethnic regions as well (Table 5).

#### Lipid profile

The mean total cholesterol of the study population was found to be normal among the studied population; however, significant elevation (*p*<*0.05*) was noted in the expatriates. Besides, expatriates from the MENA region showed significantly elevated total cholesterol levels over those of the South Asia. Triglycerides were significantly lower in Saudi nationals (*p*<*0.05*). It was below the critical level; whereas, the mean triglycerides level was higher in expatriates and higher than the critical level. Also, there was a significant association between the South Asians and MENA region population (*p*<*0.05*). Interestingly, also there was an observed elevated total cholesterol/HDL, LDL/HDL, and triglycerides/HDL ratios in the expatriates with significant difference between those of the MENA region and South Asia. These ratios were lower in Saudi populations (even lower than the critical levels) than those of expatriates. On the other hand, a significantly lower LDL was noted in the expatriates (*p*<*0.05*) than the Saudi population with significantly lower levels in expatriates from South Asia (*p*<*0.05*). Likewise for HDL: it showed a significant difference between the Saudi population and expatriates, with higher levels in the Saudi population. Meanwhile, both the MENA region and South Asia showed no significant difference between them (Table 5).

## Discussion

Non-communicable disease (NCD) is the leading cause of death in high income countries such as Saudi Arabia. Epidemiological data showed that NCD is the main reason for almost 78% of deaths in Saudi Arabia (19) with almost 46% being CVD-related deaths and its health care annual cost has been calculated to be almost $368 billion (20). The General Authority for Statistics in Saudi Arabia states that the population of Saudi Arabia in 2019 (during the research) was 34,218,169 people and the population in Tabuk in 2019 was 647,000 people (21). We found almost 18,746 patients had been diagnosed with AMI (2.9%) from the total population of the Tabuk region. So, such diseases require critical attention. Many researchers had studied the risk factors for AMI. Yet, according to our best knowledge, this is the first time the ethnicity factor has been studied as an and in relation to risk factors for such non-communicable diseases; AMI, in different ethnic populations away from their nation of origin. The care model for STEMI-diagnosed patients whether Saudi Nationals or expatriates is the same. It is already laid out by the Saudi Heart Association (https://saudi-heart.com/wp-content/uploads/2020/12/Clinical-practice-guidelines.pdf). There is no insurance barrier. According to the law of the Saudi Government, expatriates should have medical insurance from their employer or through the private sector which covers these cardiovascular diseases. There is no difference in treatment between Saudi nationals working in either governmental or private sectors. So, studying the ethnic and environmental factors can be explored with no presence of administrative or insurance problems. This study may give an indication of the importance of the environmental factors for the genetic expression for the risk factors such as diet behavior. It is worth mentioning that the studied risk factors are called the traditional risk factors for AMI. Other factors such as previously diagnosed coronary artery disease and using medications for the risk factor control in secondary prevention are called non-traditional risk factors (22). Research has reported that these non-traditional cardiac risk factors carried a negative likelihood ratio of 0.61 for the diagnosis and for the inclusion in the risk factors of AMI. Besides, patients using medications for the risk factor control in secondary prevention are non-specific factors which has been previously reported to that they limit their use as a risk factor for AMI. So, our studied risk factors are the main factors for analysis of the ethnic factor in these main risk factors. Moreover, some commonly used therapies such as statins, anti-hypertensives, anti-diabetics, and anti-coagulants are not included in this study for risk factors of AMI. We believe this would make this study more complicated than necessary. Meanwhile, we will investigate the influence of current medication history and medication adherence in cardiovascular diseases for our longitudinal studies in near future.

In the present study, nearly half of the studied population from Saudi Arabia had a past medical history of both diabetes (46.78%) and hypertension (44.69%) which are considered predominant risk factors of acute myocardial infarction (AMI). These findings substantiate those findings of other investigations conducted in the Saudi population (23, 24). Male gender is also one of the risk factors which is already established in previous studies (25, 26). Although, it has been previously reported that women were more susceptible to chronic diseases and atherosclerotic cardiovascular disease in Riyadh city (20). This indicates that environmental factors such as nutrition can be one of the reasons for such variation. Nutritional habits differ between the Tabuk and Riyadh regions. Moreover, this gives an indication that pharmacists should focus on raising awareness among male through using group teaching for males, direct procures for males, etc. in the Tabuk region. Other risk factors including age >50 years and smoking status in this study also substantiated by the other study conducted in the northern region of Saudi Arabia (27, 28). However, it has been reported that 83.33% of the studied population had a history of dyslipidemia (27). In contrast, the present study showed that Saudi patients had normal mean tFotal cholesterol, triglycerides, TC/HDL, LDL/HDL, and TG/HDL ratios with significant association with the expatriates. The genetic contribution for such variation has been proved by the significant difference between expatriates from the MENA region and South Asia. Conversely, the mean LDL level and HDL were more than the optimal level with a significant association with the expatriates. Meanwhile, HDL showed no significant association between expatriates from the MENA region and South Asia. This indicates that the genetic factor may not contribute to such variations. In other words, environmental factors such as diet behavior may contribute to such variation in HDL levels. This difference can be explained by drinking Arabian coffee and eating dates. It has been proved that drinking Arabian coffee has elevating effect on HDL and Arabian coffee drinkers have higher HDL compared to those who consume less Arabian coffee (29). Besides, it has been proved that the consumption of dates has a great effect on the lipid profile (30). Saudi nationals regularly consume Arabian coffee and Date fruit much more than expatriates in their daily life. A recent cross-sectional study conducted in Saudi Arabia addressed poor awareness of CAD risk factors irrespective of nationality, sex, level of education, marital status, employment status, income, etc. (31). Additionally, the awareness is poorer in the Saudi population (32, 33) than in the MENA region population including Lebanon (34) and Oman (35). Spreading essential awareness among the public is warranted to minimize the incidence and mortality due to CAD in developing countries including Saudi Arabia (33).

Although the Saudi population has no significant association with the lipid profile in the present study, lipid profile is a predominant risk factor among expatriates, especially from the MENA region.The Saudi population has lower TC, TG, TC/HDL, TG/HDL, and LDL/HDL than the expatriates. These associations were statistically significant with statistically significantly higher values in the MENA region than in South Asia. Nevertheless, our study findings are consistent with the previous findings including that dyslipidemia is one of the important risk factors for myocardial infarction in the South Asian population (36), especially hypertriglyceridemia (37), low-HDL level (38) and elevated LDL (39), (39). Furthermore, poor glycaemic control in the known case and impaired glucose tolerance in normal individuals among South Asians (40) due to the high prevalence of insulin resistance (41) which increases the risk of cardiovascular events (36, 37). The elevated TC/HDL and TG/HDL ratio among the South Asians and MENA region population in the present investigation authenticates the recently reported research as TC/HDL (38) and TG/HDL (39) ratio found to have the predominant risk factors for AMI. Moreover, these ratios are significantly worse in the MENA region population than in South Asians. In addition, smoking history, poor glycaemic status, and uncontrolled blood pressure are found to be other risk factors for South Asians. In the present study, the expatriates from the MENA region belong to low-middle income countries,; while Saudi Arabia is considered as a high-income country in this region (3). Among the low-middle income region hypercholesterolemia and diabetes highly contributed to MI in the low-income countries in the MENA region (40). Poor awareness of cardiovascular risk factors in both South Asian countries and the MENA region including India, Pakistan, Bangladesh, and Egypt (39) contributes to the high prevalence of MI in this studied population. Moreover, we considered only admitted cases with AMI due to the availability of laboratory diagnostic analysis records. Yet, setting the control of the ethnic groups who were not affected by such risk factors is a challenging issue for future studies. Awareness of Cardiovascular risk factors also should be emphasized by the health care professionals to the patients. Obesity is another important risk factor for MI not being investigated which is considered a limitation. Nonetheless, abdominal obesity measuring with waist-to-hip ratio has a significant positive correlation with MI; whereas, body mass index was found to have a weak correlation in this regard (41).

## Conclusion

Acute Myocardial infarction is a threat to the population irrespective of their ethnic region. However, the risk factor may be variable according to the ethnicity of the study population. Healthcare care professionals should take this factor into their consideration while educating high-risk patients with acute myocardial infarction.

## Data availability statement

The original contributions presented in the study are included in the article/Supplementary material, further inquiries can be directed to the corresponding author/s.

## Ethics statement

The study protocol was approved by the Local Research Ethics Committee of the University of Tabuk under the number (UT-077-019-014) and, including the use of oral consent for the collection of data. Moreover, the study got the Institutional Review Board (IRB) from the General Directorate of Health Affairs, Tabuk region (Registration no H-07-UT-077). The Committee unconditionally approved the project and agreed to verbal consent to be used. The Ethics Committee waived the requirement of written informed consent for participation.

## Author contributions

UA, HA, ATSA, SD, and AAA contributed by the collection of data. PA contributed his intellectual ability to the conception of the project, design, and drafting the article. TA and SZ did the statistical analysis. AA performed the data interpretation. AH contributed to the final version of the manuscript. All authors contributed to the article and approved the submitted version.

## Conflict of interest

Author ATSA was employed by the Al-Dawaa Medical Services Company. The remaining authors declare that the research was conducted in the absence of any commercial or financial relationships that could be construed as a potential conflict of interest.

## Publisher's note

All claims expressed in this article are solely those of the authors and do not necessarily represent those of their affiliated organizations, or those of the publisher, the editors and the reviewers. Any product that may be evaluated in this article, or claim that may be made by its manufacturer, is not guaranteed or endorsed by the publisher.

## Abbreviations

ACE: Africa middle east cardiovascular epidemiological study
AMI: acute myocardial infarction
CHD: coronary heart disease
CICU: coronary intensive care unit
DALYs: disability-adjusted life years lost
HDL: high density lipoprotein
LDL: low density lipoprotein
MENA: middle east north Africa
NCD: non-communicable disease
NCEP ATP III: national cholesterol education program adult treatment panel III
TC: total cholesterol
TG: triglyceride.

## References

[1] The World Bank World Development Indicator Database. Available online at: https://data.worldbank.org/country/saudi-arabia?view=chart (accessed April 13, 2022).

[2] Institute of Health Metrics Evaluation. (2022). Available online at: https://www.healthdata.org/saudi-arabia (accessed April 13, 2022).

[3] Vollset SE Goren E Yuan CW Cao J Smith AE Hsiao T. Fertility, mortality, migration, and population scenarios for 195 countries and territories from 2017 to 2100: a forecasting analysis for the global burden of disease study. Lancet. (2020) 396:1285–306. 10.1016/S0140-6736(20)30677-2 32679112 PMC7561721

[4] Baghdadi LR Alhassan MK Alotaibi FH AlSelaim KB Alzahrani AA AlMusaeed FF. Anxiety, depression, and common chronic diseases, and their association with social determinants in saudi primary care. J Prim Care Community Health. (2021) 12:21501327211054987. 10.1177/21501327211054987 34814776 PMC8673869

[5] Kingdom of Saudi Arabia: National Transformation Program. (2020). Available online at: https://www.vision2030.gov.sa/v2030/vrps/ntp/ (accessed April 13, 2022).

[6] The Global Status Report on Non communicable Diseases. (2020). Available online at: https://www.who.int/publications/i/item/9789240000490 (accessed April 13, 2022).

[7] Michael JP Ann MN Daniel W Robert JS Irfan K Joseph E. Quantifying importance of major risk factors for coronary heart disease. Circulation. (2019) 139:1603–11. 10.1161/CIRCULATIONAHA.117.031855 30586759 PMC6433489

[8] Hassannejad R Mansourian M Marateb H Mohebian MR Gaziano TA Jackson RT. Developing non-laboratory cardiovascular risk assessment charts and validating laboratory and non-laboratory-based models. Glob Heart. (2021) 16:58. 10.5334/gh.890 34692382 PMC8428313

[9] The total population in 2021. General Authority for Statistics (Saudi Arabia) and National Code of Countries and Nationalities. Available online at: https://www.stats.gov.sa/en/saudi-standard-classification-of-occupations (accessed April 13, 2022).

[10] Migration Profile: Saudi Arabia,. "Unicef, United Nations. (2021). Available online at: https://esa.un.org/miggmgprofiles/indicators/files/saudiarabia.pdf (accessed April 13, 2022).

[11] Ahmed AM Hersi A Mashhoud W Arafah MR Abreu PC Al Rowaily MA. Cardiovascular risk factors burden in Saudi Arabia: the Africa middle east cardiovascular epidemiological (ACE) study. J Saudi Heart Assoc. (2017) 29:235–43. 10.1016/j.jsha.2017.03.004 28983166 PMC5623029

[12] Al-Ghamdi S Alzubaidi FK Alharthai SA Alzahim MS Al Bahily FM Alsifaee MI. Prevalence and correlates of diastolic dysfunction in patients with hypertension: a cross-sectional study from in the Kingdom of Saudi Arabia. Pan Afr Med J. (2021) 40:159. 10.11604/pamj.2021.40.159.31089 34970401 PMC8683461

[13] Monestime S Beech B Kermah D Norris K. Prevalence and predictors of obesity-related cancers among racial/ethnic groups with metabolic syndrome. PLoS ONE. (2021) 16:e0249188. 10.1371/journal.pone.0249188 33826671 PMC8026066

[14] Alhabib KF Kinsara AJ Alghamdi S Al-Murayeh M Hussein GA AlSaif S. The first survey of the saudi acute myocardial infarction registry program: main results and long-term outcomes (STARS-1 Program). PLoS ONE. (2019) 14:e0216551. 10.1371/journal.pone.0216551 31112586 PMC6528983

[15] Messerli FH Rimoldi SF Bangalore S. Changing definition of hypertension in guidelines: how innocent a number game? Eur Heart J. (2018) 39:2241–2. 10.1093/eurheartj/ehx806 29324999

[16] Powers MA Bardsley JK Marjorie Cypress Funnell MM Harms D Hess-Fischl A Hooks B. Diabetes self-management education and support in adults with type 2 diabetes: a consensus report of the American diabetes association, the association of diabetes care & education specialists, the academy of nutrition and dietetics, the American academy of family physicians, the American academy of PAs, the American association of nurse practitioners, and the American pharmacists association. Diabetes Care. (2020) 43:1636–49. 10.2337/dci20-0023 32513817

[17] Yu KH Chen HH Cheng TT Jan YJ Weng MY Lin YJ. Consensus recommendations on managing the selected comorbidities including cardiovascular disease, osteoporosis, and interstitial lung disease in rheumatoid arthritis. Medicine. (2022) 101:e28501. 10.1097/MD.0000000000028501 35029907 PMC8735742

[18] Zhou H Zhang S Sun X Yang D Zhuang X Guo Y. Lipid management for coronary heart disease patients: an appraisal of updated international guidelines applying appraisal of guidelines for research and evaluation II-clinical practice guideline appraisal for lipid management in coronary heart disease. J Thorac Dis. (2019) 11:3534–46. 10.21037/jtd.2019.07.71 31559060 PMC6753419

[19] Al-Hanawi MK. Socioeconomic determinants and inequalities in the prevalence of non-communicable diseases in Saudi Arabia. Int J Equity Health. (2021) 20:174. 10.1186/s12939-021-01510-6 34321000 PMC8320210

[20] AlQuaiz AM Kazi A Alodhayani AA Almeneessier A AlHabeeb KM Siddiqui AR. Age and gender differences in the prevalence of chronic diseases and atherosclerotic cardiovascular disease risk scores in adults in Riyadh city, Saudi Arabia. Saudi Med J. (2021) 42:526–36. 10.15537/smj.2021.42.5.20200684 33896782 PMC9149694

[21] General Authority of Statistic. Available online at: https://www.stats.gov.sa/en/43 (accessed April 13, 2022).

[22] Body R McDowell G Carley S Mackway-Jones K. Do risk factors for chronic coronary heart disease help diagnose acute myocardial infarction in the emergency department? Resuscitation. (2008) 79:41–5. 10.1016/j.resuscitation.2008.06.009 18691797

[23] Al-Shehri AM. Prevalence and pattern of lipid disorders in Saudi patients with angiographically documented coronary artery disease. J Family Community Med. (2014) 21:166–9. 10.4103/2230-8229.142970 25374467 PMC4214005

[24] Abazid R Al Saqqa H Smettei O. Analysis of three risk stratification systems in a Saudi population. J Saudi Heart Assoc. (2017) 29:96–101. 10.1016/j.jsha.2016.06.002 28373783 PMC5366669

[25] Alhabib KF Hersi A Alfaleh H Alnemer K Alsaif S Taraben A. Baseline characteristics, management practices, and in-hospital outcomes of patients with acute coronary syndromes: results of the Saudi project for assessment of coronary events (SPACE) registry. J Saudi Heart Assoc. (2011) 23:233–9. 10.1016/j.jsha.2011.05.004 23960654 PMC3727434

[26] Bentley-Lewis R Aguilar D Riddle MC Claggett B Diaz R Dickstein K. Rationale, design, and baseline characteristics in evaluation of LIXisenatide in acute coronary syndrome, a long-term cardiovascular end point trial of lixisenatide versus placebo. Am Heart J. (2015) 169:631–8.e7. 10.1016/j.ahj.2015.02.002 25965710

[27] Alhassan SM Ahmed HG Almutlaq BA Alanqari AA Alshammari RK Alshammari KT. Risk factors associated with acute coronary syndrome in northern Saudi Arabia. In search of a perfect outfit. J Cardiol Curr Res. (2017) 8:00281. 10.15406/jccr.2017.08.00281

[28] Yagoub U Saiyed NS Al Qahtani B Al Zahrani AM Birema Y Al Hariri I. Investigating the incidence and risk factors of hypertension: a multicentre retrospective cohort study in Tabuk, Saudi Arabia. PLoS ONE. (2022) 17:e0262259. 10.1371/journal.pone.0262259 34990492 PMC8735626

[29] Alfawaz HA Khan N Yakout SM Khattak MNK Alsaikhan AA Almousa AA. Prevalence, predictors, and awareness of coffee consumption and its trend among saudi female students. Int J Environ Res Public Health. (2020) 17:7020. 10.3390/ijerph17197020 32992846 PMC7579070

[30] Alalwan TA Perna S Mandeel QA Abdulhadi A Alsayyad AS D'Antona G. Effects of daily low-dose date consumption on glycemic control, lipid profile, and quality of life in adults with pre- and type 2 diabetes: a randomized controlled trial. Nutrients. (2020) 12:217. 10.3390/nu12010217 31952131 PMC7019638

[31] Almalki MA AlJishi MN Khayat MA Bokhari HF Subki AH Alzahrani AM. Population awareness of coronary artery disease risk factors in Jeddah, Saudi Arabia: a cross-sectional study. Int J Gen Med. (2019) 12:63–70. 10.2147/IJGM.S184732 30666149 PMC6333320

[32] Enani S Bahijri S Malibary M Jambi H Eldakhakhny B Al-Ahmadi J. The association between dyslipidemia, dietary habits and other lifestyle indicators among non-diabetic attendees of primary health care centers in jeddah, Saudi Arabia. Nutrients. (2020) 12:2441. 10.3390/nu12082441 32823801 PMC7469008

[33] Basham K Aldubaikhi A Sulaiman I Alhaider A Alrasheed A Bahanan F. Public awareness of early symptoms of acute myocardial infarction among Saudi population. J Family Med Prim Care. (2021) 10:3785–90. 10.4103/jfmpc.jfmpc_449_21 34934681 PMC8653441

[34] Fahs I Khalife Z Malaeb D Iskandarani M Salameh P. The prevalence and awareness of cardiovascular diseases risk factors among the lebanese population: a prospective study comparing urban to rural populations. Cardiol Res Pract. (2017) 2017:3530902. 10.1155/2017/3530902 28465858 PMC5390633

[35] Ammouri AA Tailakh A Isac C Kamanyire JK Muliira J Balachandran S. Knowledge of coronary heart disease risk factors among a community sample in oman: pilot study. Sultan Qaboos Univ Med J. (2016) 16:e189–96. 10.18295/squmj.2016.16.02.009 27226910 PMC4868518

[36] Saha SP Banks MA Whayne TF. Managing cardiovascular risk factors without medications: what is the evidence? Cardiovasc Hematol Agents Med Chem. (2021) 19:8–16. 10.2174/1871525718666200518093418 32418531

[37] Mathew A Hong Y Yogasundaram H Nagendran J Punnoose E Ashraf SM. Sex and medium-term outcomes of ST-segment elevation myocardial infarction in Kerala, India: a propensity score-matched analysis. CJC Open. (2021) 3:S71–80. 10.1016/j.cjco.2021.09.023 34993436 PMC8712709

[38] Makshood M Post WS Kanaya AM. Lipids in South Asians: epidemiology and management. Curr Cardiovasc Risk Rep. (2019) 13:24. 10.1007/s12170-019-0618-9 33833849 PMC8026164

[39] Mahmood S Jalal Z Hadi MA Khan TM Haque MS Shah KU. Prevalence of non-adherence to antihypertensive medication in Asia: a systematic review and meta-analysis. Int J Clin Pharm. (2021) 43:486–501. 10.1007/s11096-021-01236-z 33515135

[40] Li Y Chen X Li S Ma Y Li J Lin M. Non-high-density lipoprotein cholesterol/high-density lipoprotein cholesterol ratio serve as a predictor for coronary collateral circulation in chronic total occlusive patients. BMC Cardiovasc Disord. (2021) 21:311. 10.1186/s12872-021-02129-9 34162320 PMC8223315

[41] Medhat M Sabry N Ashoush N. Knowledge, attitude and practice of community pharmacists towards nutrition counseling. Int J Clin Pharm. (2020) 42:1456–68. 10.1007/s11096-020-01106-0 32860597

